# Systematic review on use, cost and clinical efficacy of automated decontamination devices

**DOI:** 10.1186/s13756-021-00894-y

**Published:** 2021-02-12

**Authors:** Stephanie J. Dancer, Marco-Felipe King

**Affiliations:** 1 Department of Microbiology, Hairmyres Hospital, NHS, Lanarkshire, G75 8RG Scotland, UK; 2 School of Applied Sciences, Edinburgh Napier University, Edinburgh, Scotland, UK; 3 School of Civil Engineering, University of Leeds, Leeds, UK

**Keywords:** Decontamination, Environment, Hospital-acquired infection, Ultraviolet light, Hydrogen peroxide, Toxicity, Cost

## Abstract

**Background:**

More evidence is emerging on the role of surface decontamination for reducing hospital-acquired infection (HAI). Timely and adequate removal of environmental pathogens leads to measurable clinical benefit in both routine and outbreak situations.

**Objectives:**

This systematic review aimed to evaluate published studies describing the effect of automated technologies delivering hydrogen peroxide (H202) or ultra-violet (UV) light on HAI rates.

**Methods:**

A systematic review was performed using relevant search terms. Databases were scanned from January 2005 to March 2020 for studies reporting clinical outcome after use of automated devices on healthcare surfaces. Information collected included device type, overall findings; hospital and ward data; study location, length and size; antimicrobial consumption; domestic monitoring; and infection control interventions. Study sponsorship and duplicate publications were also noted.

**Results:**

While there are clear benefits from non-touch devices in vitro, we found insufficient objective assessment of patient outcome due to the before-and-after nature of 36 of 43 (84%) studies. Of 43 studies, 20 (47%) used hydrogen peroxide (14 for outbreaks) and 23 (53%) used UV technology (none for outbreaks). The most popular pathogen targeted, either alone or in combination with others, was *Clostridium difficile* (27 of 43 studies: 63%), followed by methicillin-resistant *Staphylococcus aureus* (MRSA) (16 of 43: 37%). Many owed funding and/or personnel to industry sponsorship (28 of 43: 65%) and most were confounded by concurrent infection control, antimicrobial stewardship and/or cleaning audit initiatives. Few contained data on device costs and rarely on comparable costs (1 of 43: 2%). There were expected relationships between the country hosting the study and location of device companies. None mentioned the potential for environmental damage, including effects on microbial survivors.

**Conclusion:**

There were mixed results for patient benefit from this review of automated devices using H202 or UV for surface decontamination. Most non-outbreak studies lacked an appropriate control group and were potentially compromised by industry sponsorship. Concern over HAI encourages delivery of powerful disinfectants for eliminating pathogens without appreciating toxicity or cost benefit. Routine use of these devices requires justification from standardized and controlled studies to understand how best to manage contaminated healthcare environments.

## Introduction

Hospital-based cleaning and disinfection of environmental surfaces is now recognised as a crucial component of infection prevention and control [1]. This has generated interest in a range of automated decontamination technologies over the past decade. So-called ‘no-touch’ devices disperse chosen microbiocidal products into the healthcare environment in order to disinfect surfaces and reduce the risk of hospital-acquired infection (HAI) [2]. Given that the agents utilised by these devices are toxic to humans, the mobile equipment designed to deliver them are necessarily controlled through remote access. Chemical products include chlorine dioxide, hydrogen peroxide and ozone in gaseous form; and peracetic acid, quaternary ammonium compounds and hydrogen peroxide as aerosols [1–3]. Another compound, peroxone, combines hydrogen peroxide and ozone to create a dual system with enhanced oxidation [4]. Alternative decontamination technology makes use of ultra-violet (UV) light, which is produced  using either mercury or xenon bulbs [1–3]. There are multiple reports describing the in vitro effect of these systems, but studies investigating the clinical impact on hospital patients tend to favour devices dispelling either hydrogen peroxide or UV light. It was decided to review these specific technologies in order to further examine their effects on clinical benefit and cost.

Most UV devices use low-pressure mercury gas bulbs to generate UV-C light with a targeted wavelength of 254 nm; pulsed xenon devices produce a broader spectrum of UV light in short pulses with a target wavelength of 200–315 nm [3]. UV light breaks DNA bonds resulting in death of microorganisms, including spores [5]. Aerosolized hydrogen peroxide systems utilize 3–7% hydrogen peroxide, sometimes with silver ions, with particle sizes ranging from 2 to 12 μm [6]. These particles are released into a room, followed by passive aeration. The vapour system is based on micro-condensation, which allows the vaporization of concentrated (30–35%) hydrogen peroxide under controlled humidity [6].

While there is no doubt over the in vitro capacity of these technologies to eliminate surface pathogens, there are concerns over practicalities of use, toxicity and cost-benefits in vivo [7, 8]. Not all studies report complete removal of specific pathogen reservoirs or, indeed, significant reductions in HAI rates. It is clear that prior removal of dirt is essential before deployment of these devices [9–11]. There is additional concern over the data provided since selective reporting from quasi-experimental studies does not necessarily offer a balanced view of the technology evaluated [12]. Some studies are conflicted by ongoing or newly introduced infection prevention or cleaning interventions during the study period; others report, or fail to report, concurrent or newly introduced antimicrobial stewardship initiatives [13, 14]. Many studies omit mention of environmental monitoring, cleaning efficacy or even baseline cleaning protocols [7, 14]. Furthermore, studies using these devices do not necessarily disclose sources of funding or declare potential conflicts from industry sponsorship, including the provision of training, equipment, report writing and/or personnel [1, 15].

The aim of this systematic review is to critically assess study design, confounders, costs and overall clinical outcome for decontamination devices using hydrogen peroxide or UV light for surfaces in the healthcare environment. Given the recent increase in use of these devices, it is timely to offer objective comments on real life impact for patients and healthcare budgets [7].

## Methods

**Fig. 1:**
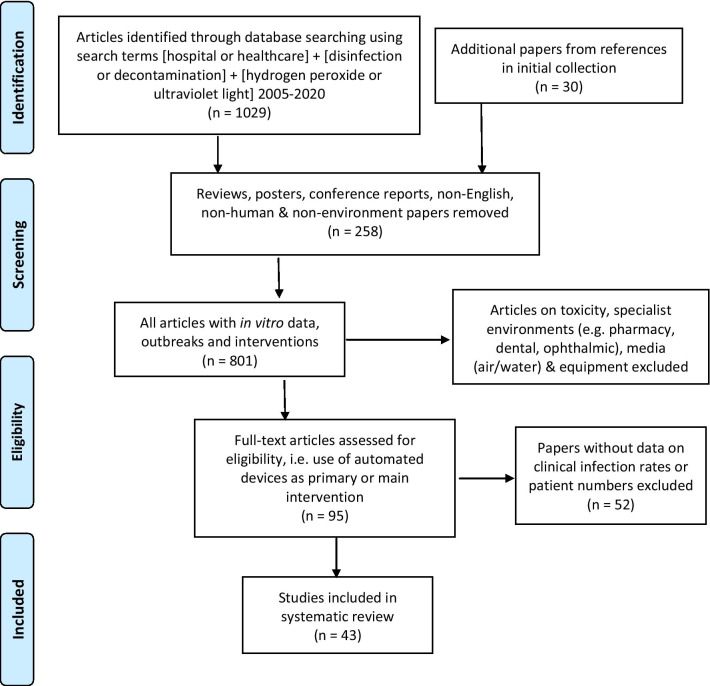
PRISMA 2009 Flow Diagram for selecting papers for a systematic review on use, cost and clinical efficacy of automated decontamination devices

A systematic review was performed using relevant search terms: [hospital or healthcare] + [disinfection or decontamination] + [hydrogen peroxide or ultraviolet light] (Fig. 1). The databases employed were PubMed, CINAHL, CDSR, DARE and EMBASE from Jan 2005 to March 2020 for studies evaluating automated device technology using ultraviolet microbiocidal light (UV) or hydrogen peroxide (H202) in healthcare facilities for contamination on surfaces ± air using indicator pathogens ± aerobic colony counts; cost of technology; and HAI rates for *Clostridium difficile*, methicillin-resistant *Staphylococcus aureus* (MRSA), vancomycin-resistant enterococci (VRE), coliforms (*Escherichia coli*, *Klebsiella pneumoniae*, *Enterobacter* spp., *Serratia* spp.), *Pseudomonas* spp., *Acinetobacter* spp., and generic multidrug-resistant organisms (MDROs) including extended-spectrum-beta-lactamase producing coliforms (ESBLs). Studies describing in vitro; in situ; experimental and/or surface effects of non-touch technologies without concurrent data on patient impact were scanned before exclusion, along with non-English papers, posters and conference reports (Fig. 1). Hospital location, types, study ward/unit, study length and size, antimicrobial consumption (total; specific classes); domestic monitoring, infection control interventions and other variables that might impact on the results were noted. Specialist healthcare environments such as outpatients, pharmacies and clean rooms were excluded, as were articles describing microbiological impact of automated devices on specific items of equipment. Published data was checked for duplicate or linked publications, funding, sponsors and industry involvement if acknowledged.

### Statistical analysis

Statistical review and analysis was applied to common data presented in selected device papers. Risk of bias was assessed for each study by evaluating study design, methodological consistency, infection control confounders, population and study unit heterogeneity, sampling bias, outcome evaluation, involvement of sponsor and selective reporting. Power calculations and overall numbers were examined against reported significance values.

## Results

### Study demographics

**Fig. 2:**
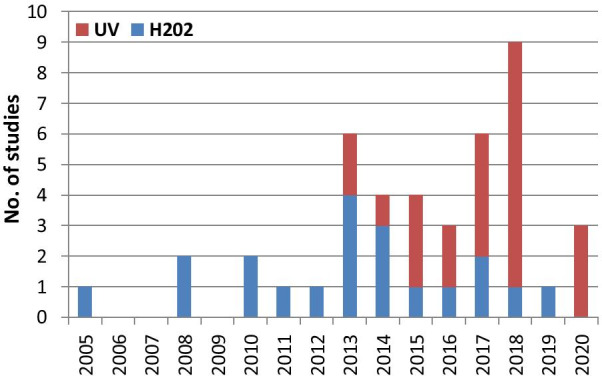
Year of study (2005–2020) and device type (UVC vs H202) over review period

**Fig. 3:**
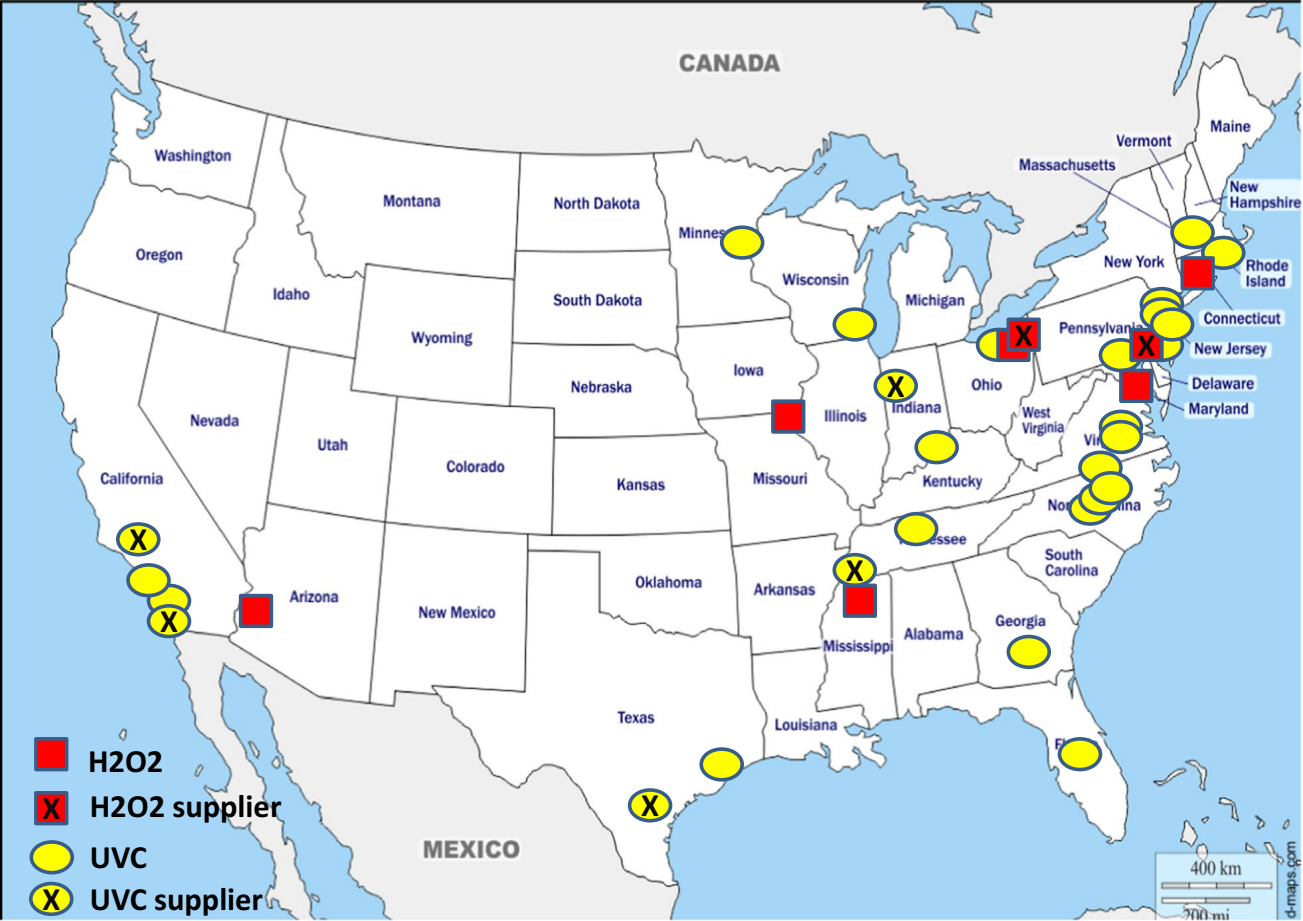
Location of UV and H202 industries and study institutions in the USA

**Fig. 4:**
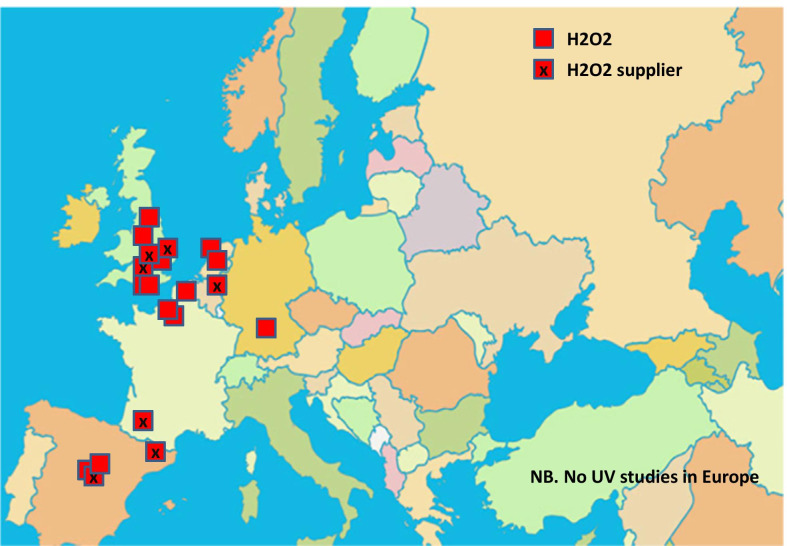
Location of H202 industries and study institutions in Europe

There were a total of 43 studies presenting the effects on HAI rates from automated devices delivering either hydrogen peroxide or UV light (Table 1; Figs. 1, 2) [16–58]. These were published between 2005 and 2020 and include brief reports and letters describing either intervention or outbreak control studies involving different hospitals from eight countries in the developed world. Of these, 20 (47%) used some form of hydrogen peroxide delivery (14 for outbreaks) and 23 (53%) used UV technology (none for outbreaks). Over half the papers (28; 65%) originated from the USA, with six describing the effects of hydrogen peroxide devices [37, 38, 47, 49, 55, 57] and 22 utilising UV technology (Fig. 3) [16, 18, 21–31, 34–36, 39–41, 43, 48, 50]. Five (12%) studies were based in the UK, all using hydrogen peroxide, [32, 44, 53, 56, 58] and three (7%) others using hydrogen peroxide were performed in France (Fig. 4) [45, 46, 51]. Two (5%) studies each using hydrogen peroxide originated from Spain [19, 33] and The Netherlands, [20, 54] and there was one (2%) study each from Japan (UV) [17], Poland (hydrogen peroxide) [52] and Tasmania (hydrogen peroxide) [42]. There were no studies reporting use of UV in Europe.

**Table 1:**
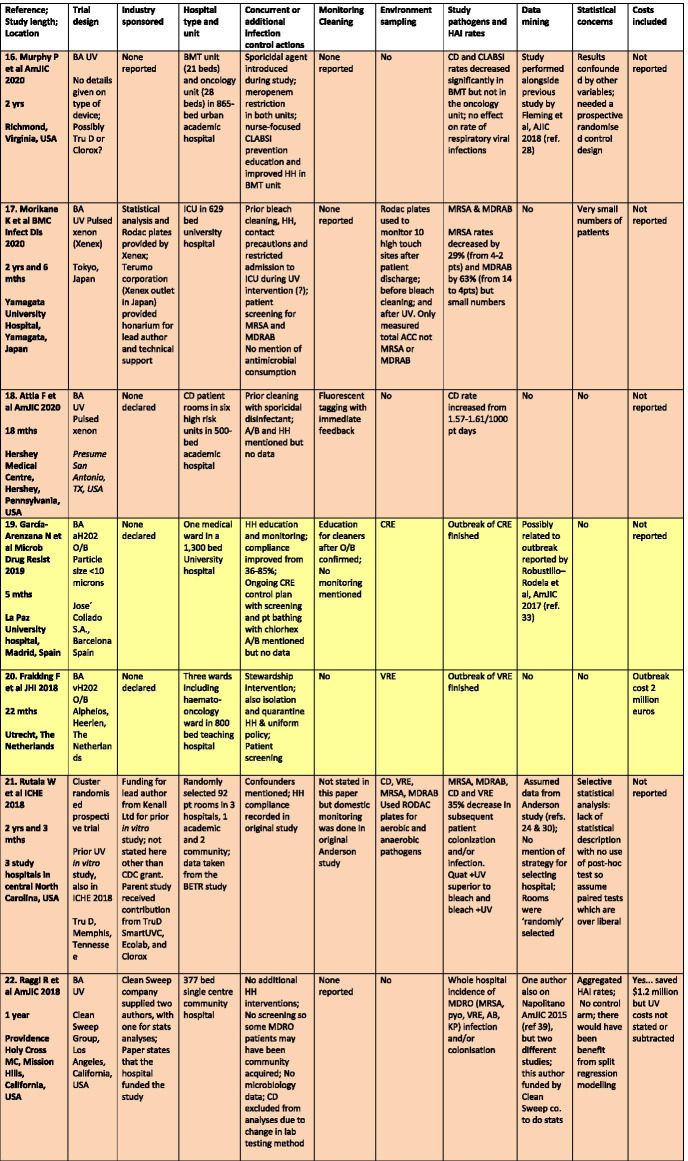

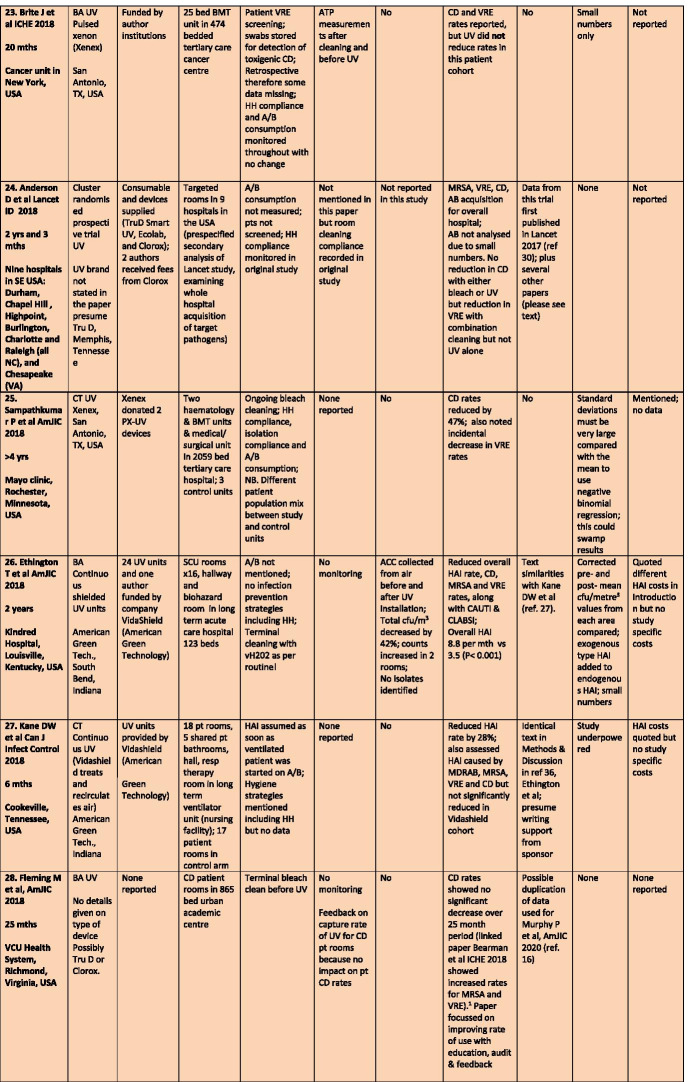

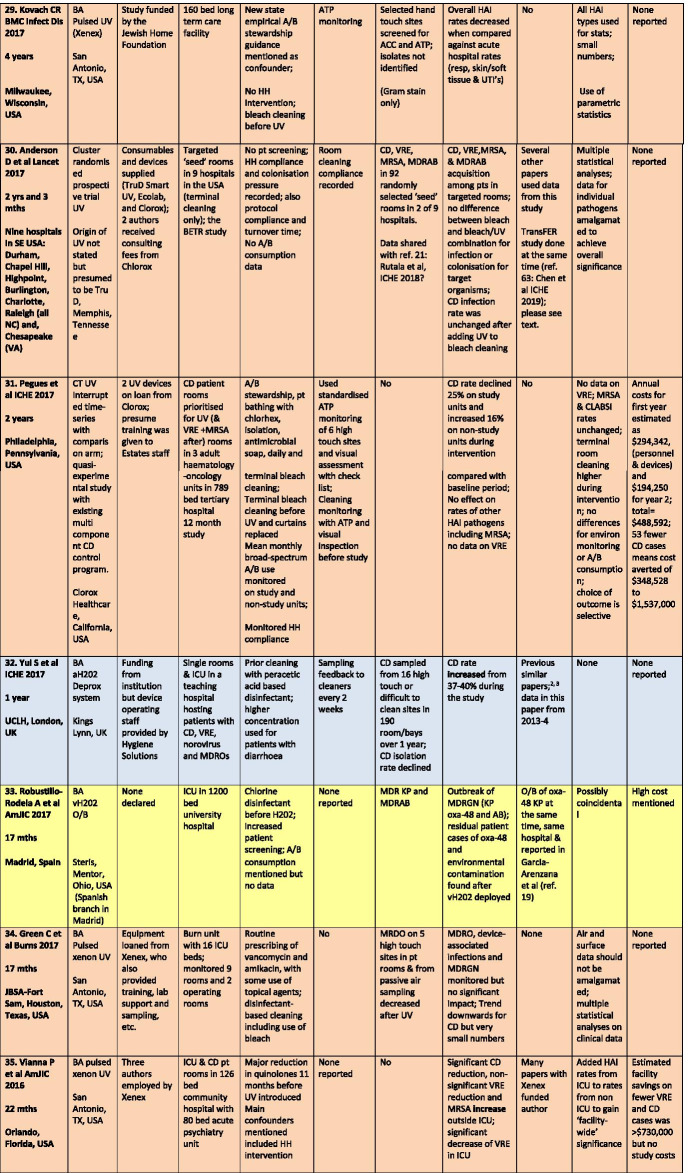

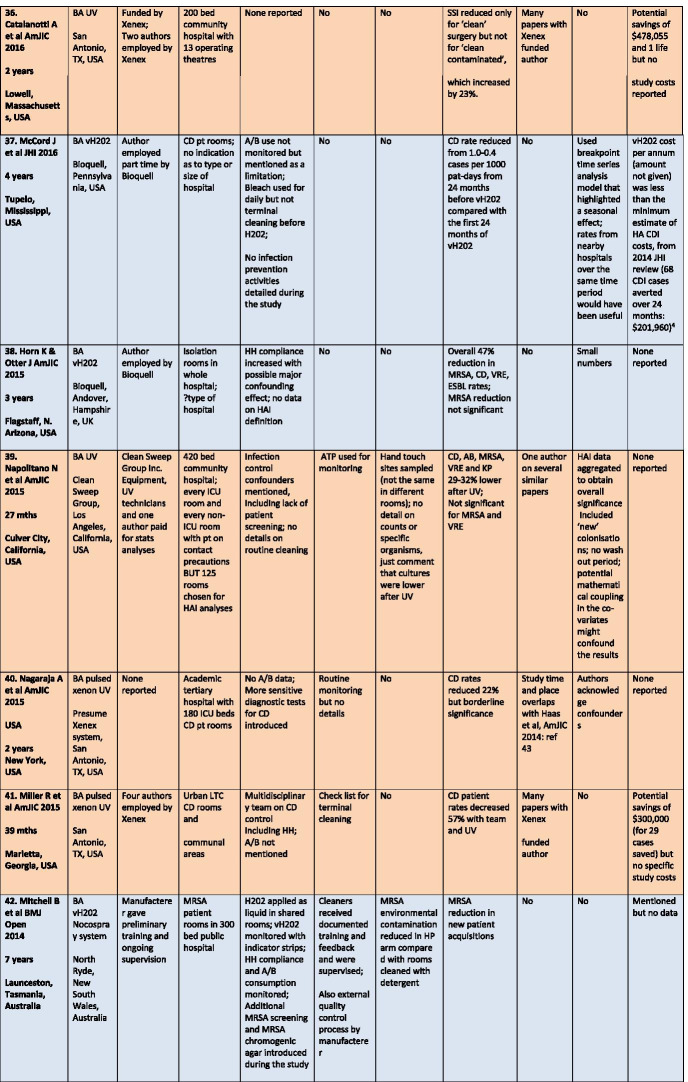

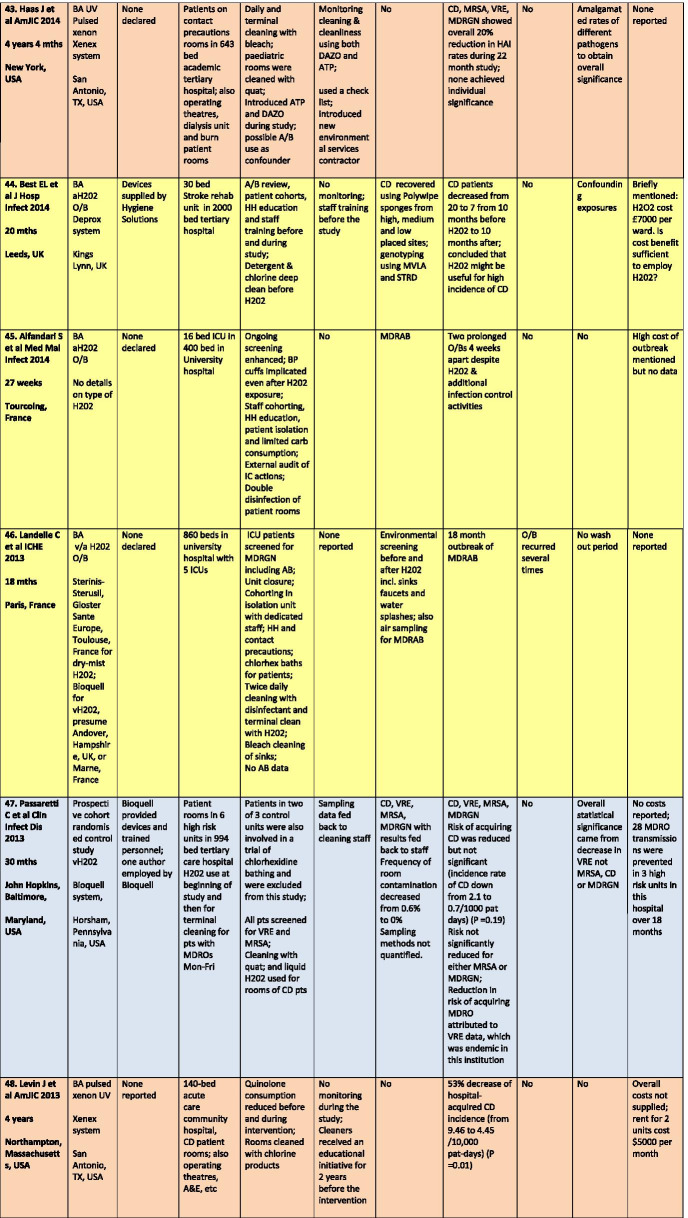

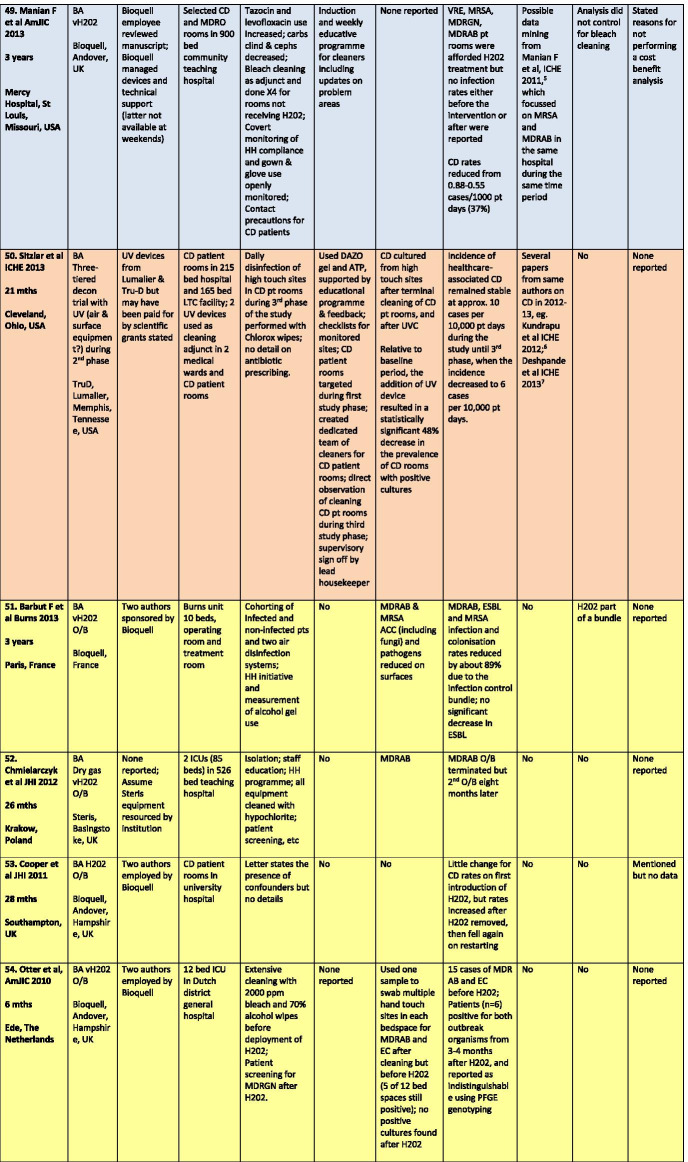

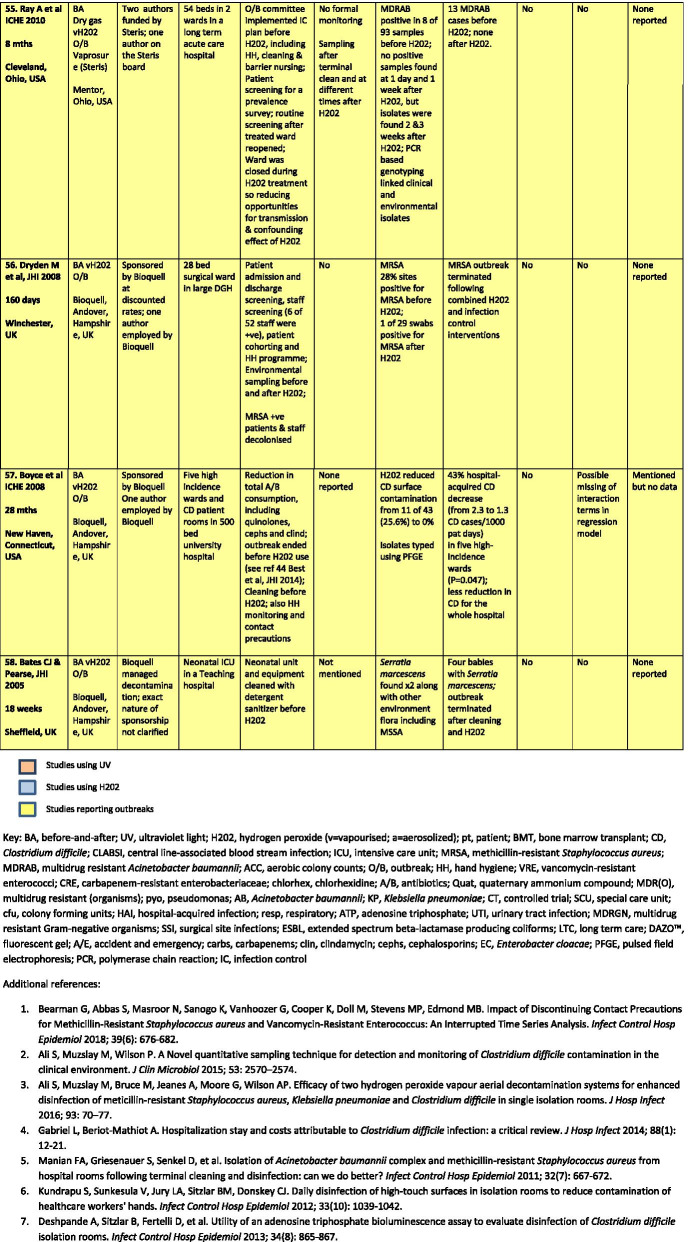
Data from 43 original or outbreak studies reporting patient benefit from use of UV or H202 automated devices

Most (36: 84%) were before-and-after studies, including 14 (33%) outbreak interventions [19, 20, 33, 44–46, 51–58]; three (7%) originated from the same cluster randomised prospective study (Benefits of Enhanced Terminal Room Disinfection: BETR study) [21, 24, 30] and the remaining four (9%) were controlled studies, [25, 27, 47] with one interrupted time series that included a control arm [31].

There were many different types of hospitals involved in assessing decontamination devices, mostly university and tertiary hospitals with 400–2000 beds; and some district general or community hospitals (100–400 beds) or long term care hospitals (50–170 beds). There were two studies performed exclusively in burns unit [34, 51]; these, along with others, did not indicate the type of host hospital. Other specialist units included intensive care [17, 26, 32, 33, 35, 39, 40, 45, 46, 52, 54]; neonatal [58]; biohazard [26] and treatment rooms [51]; bone marrow transplant [16, 23, 25]; haematology and/or oncology [16, 20, 25, 31]; respiratory therapy room [27]; operating theatres [36, 43, 48]; dialysis unit [43]; stroke rehabilitation [44]; accident and emergency [48]; and three studies mentioned bathrooms [27] and/or communal areas [26, 27, 41].

Figure 2 shows the year of publication for each study. From 2005 to 2014, most of the studies employed hydrogen peroxide devices (14 of 17: 82%) whereas from 2015 to 2020, the studies predominantly focussed on UV devices (20 of 26: 77%). The total length of the studies, i.e. period of time over which data was gathered, ranged from 18 weeks to 3 years for outbreaks (n = 14), with a median time of 17–18 months and an average duration of 16 months. For intervention studies (n = 29), the total length of time ranged from 6 months to 7 years; the median time was 27 months and the average was 30 months (Table 1).

### Effect on HAI rates

All 43 studies presented an assessment of chosen technology on HAI rates, which described the effect on one specific pathogen or a combination; from *Clostridium difficile*, methicillin-resistant *Staphylococcus aureus* (MRSA), multi-drug resistant Gram-negative coliforms (MDRGN), multi-drug resistant Acinetobacter (MDRA), *Pseudomonas aeruginosa*, vancomycin-resistant enterococci (VRE), combined multiply drug resistant organisms (MDROs); or overall HAI rates; or surgical site, catheter and device infection rates (Table 1). The most popular pathogen to control with automated devices, either alone, or in combination with others, was *C. difficile* (27 of 43 studies: 63%), followed by MRSA (16 of 43: 37%); MDRA (15 of 43: 35%); VRE (14 of 43: 33%) and MDRGN (12 of 43: 28%). There were 29 of 43 studies (67%) that also performed before and after sampling of the environment for the same pathogens as monitored for patient infections.

Most studies reported either reductions in HAI rates for the study pathogen(s) or resolution of an outbreak. Some studies reported effects on one or more pathogen rates along with no change or even increases in other pathogen rates. Two studies reported an increase in *C.difficile* rates using UV and H2O2 [18, 32] and another reported static rates for *C. difficile* and VRE following UV use in a bone marrow transplant unit [23]. One analysis of the BETR study (using UV) saw a reduction only in VRE and not in rates of infection due to *C. difficile*, MRSA or Acinetobacter, although the latter numbers were so small, the effect could not be analysed [24]. Other *C. difficile* studies using UV showed no statistically significant decrease over a 25 month period, [28] while at the same time rates decreased for the bone marrow transplant unit in the same hospital; [16] another UV study reported a decline in *C. difficile* but not for other pathogens including MRSA [31]. Mixed results for UV were also found by Vianna et al., with reductions in *C. difficile* and VRE in the study ICU but increasing MRSA and static VRE rates outside the ICU [35]. One study using UV on a burn unit found no significant impact on total MDRO, device-associated infections or MDRGNs [34]. One study targeted operating theatres and measured the impact of UV on surgical site infection rates; these decreased for ‘clean’ but not for ‘clean contaminated’ surgery, which actually increased by 23% [36]. Several studies saw non-significant effects on MRSA using both H2O2 [38, 47] and UV [39], and another UV study achieved a reduction in *C. difficile* rates only after introducing a supervised cleaning team targeting hand-touch sites with bleach wipes [50]. One protracted outbreak of MDRA reoccurred despite hydrogen peroxide and additional infection control interventions [45] and another recovered the outbreak MDRA from the environment 2–3 weeks after hydrogen peroxide treatment [55].

### Common confounders

#### Infection prevention and control

Controlling an environmental decontamination study is fraught with confounders, often due to concurrent or new initiatives in infection prevention and control introduced before, or during, the study. Some authors recognised the importance of these confounders and collected additional data in order to regulate possible conflicting effects, e.g. antimicrobial consumption; hand hygiene; and patient screening. These studies usually discussed the potential impact on overall findings. Others mentioned infection control activities implemented before or during the study without providing any detail or discussion on potential impact; this may have been mandated by a bundled approach during an outbreak or lack of space in a brief publication. Some demonstrated obvious conflicts, such as lack of admission screening, antimicrobial prescribing changes, staff education programmes or use of powerful disinfectants before, or during, device deployment. These studies would have been seriously compromised by such initiatives, when such activities are already known to have a major impact of HAI rates. The studies by Raggi et al. [22] and Haas et al. [43], Ethington et al. [26], Kane et al. [27], McCord et al. [37] and Horn and Otter [38] illustrate a range of pitfalls in a decontamination assessment [59]. Conversely, the articles by Pegues et al. [31] and Brite et al. [23] are good examples of studies that attempted to control confounders.

#### Monitoring cleaning and cleanliness

Cleaning staff are very susceptible to Hawthorne effects when a research study involves sampling general surfaces in the clinical environment [60, 61]. One mechanism for controlling changes in compliance by domestic staff is to introduce some type of monitoring; either by measuring cleaning compliance using fluorescent tagging, or by evaluating cleanliness using ATP detection to assess surface levels of organic soil [7]. Other methods involve direct observation of cleaners; supervisory sign-off; check lists; feed-back; and visual monitoring [2]. The microbiological sampling performed in many studies would have had an effect on cleaning staff because this would have been difficult to blind and would have sent out strong messages regarding cleaning efficiency. If cleaning staff detect interest in the work they do, then they usually ‘up the game’ in order to alleviate any threat toward their jobs [61]. This would have impacted on overall outcome, environmental sampling data and even HAI rates.

There is little, if any, mention of this predictable psychological reaction in any of the studies in this review. Formal monitoring is, however, mentioned in 16 of 43 (37%) studies, with use of ATP and feedback to cleaning staff as the two most common methods employed (Table 1). One study introduced both ATP and fluorescent gel tagging along with a cleaning check list and new environmental services contractor [43]. Sitzlar et al. used several methods of monitoring in their study; this proved helpful, given that a newly formed cleaning team for CD patient rooms with supervisory sign off achieved the outcome sought after UV failed to eradicate hospital-acquired *C.difficile* [50].

#### Business and industry involvement

Inevitably, sponsorship issues arise in this review of automated decontamination devices. Environmental cleaning studies are often funded by manufacturers of cleaning agents or disinfection technologies and this encourages potential conflicts of interest and the introduction of real or perceived biases into the evidence base [62]. There are many forms of sponsorship available from business and industry, ranging from full or part study funding; donation or lending of equipment; device discounts; implementation and engineering technicians; scientific support including article writing and statistical analyses; industry personnel with in the research team; study supervision; free education and training; and device maintenance, among others. A total of 28 of 43 (65%) studies reported some form or other of industry support, with at least 20 of 43 (46%) studies including industry personnel in the authorship (Table 1). Among reported declarations, there were three main companies providing support and authors for 20 of 43 (46%) studies; these were Bioquell [37, 38, 47, 49, 51, 53, 54, 56–58], Xenex [17, 25, 34–36, 41], and Clorox [21, 24, 30, 31].

There is an additional sideways strategy for industry involvement in promoting device evaluation. This comes in the form of sharing authors with specific expertise for independent studies using the same brand of device. These authors are not necessarily employees of the company but may be contracted or asked to provide support such as statistical analysis, writing or laboratory testing. This was evident in studies using UV devices, in particular [22, 26, 27, 35, 36, 39, 41]. Another strategy is so-called ‘salami slicing’, whereby multiple publications are linked with one original study; this is seen with the BETR study, which generated several papers examining whole or partial datasets using different objectives and/or perspectives for analyses [21, 24, 30, 63–65]. While none of these approaches necessarily challenge long held editorial standards on plagiarism, they could inflict bias from subtle advertising. Multiple papers from one set of data skew any future metanalyses on efficacy and hence generate uncertainty for scientists, clinical staff and policy makers [66]. Furthermore, Figs. 3 and 4 highlight the geographical relationships between the country of publication and home location of industry supplying the technology; this may well be obvious, but could add bias by encouraging similar studies from one or two countries dominating the literature.

#### Data on costs

Very few of the studies in this review offered tangible data on costs. There was no mention of any resources required or cost savings for 24 of 43 (56%) studies, with 8 (19%) giving brief mention of the importance of cost benefit without specific data [25–27, 33, 42, 45, 53, 57]. Ten (23%) studies offered incomplete costings, mostly based on potential savings from cases averted but lacking a balance against costs of the technology used [20, 22, 35–37, 41, 44, 47–49]. There was just one study (2%) that provided a complete breakdown of costs incurred alongside costs saved from cases of *C.difficile* [31]. It is not possible to compare the costs of an outbreak or HAI against overall costs of devices, maintenance, technical needs and implementation without robust data on expenses; healthcare managers need to know the full range of cost benefits when considering all options for infection prevention.

#### Statistical aberrations

Comments on statistical findings are shown in Table 1. The most important finding for non-outbreak studies was use of aggregated data that leads to the conclusion that the author wishes  to report. This is called Simpson’s paradox, where a trend appears to be positive when the data is aggregated but negative when it is disaggregated (when examining individual groups or data collections). Several studies amalgamated selected outcomes together in order to achieve statistical significance [21, 22, 26, 29, 30, 34, 35, 39, 43, 47]. This clearly skews reporting and future conclusions from metanalyses.

There was also selective reporting, in that some pathogen rates were monitored but no outcome data was offered. Pegues et al. did not provide any VRE data despite using UV devices for VRE patients; Morikane et al. only measured total ACCs and not MRSA or MDRAB from environmental sampling, despite measuring MRSA and MDRAB HAI rates [17, 31].

Several papers presented statistical analysis which were either underpowered [27] or were performed on small numbers of patients [17, 23, 26, 29, 38]; two papers openly acknowledged statistical limitations [24, 40].

## Discussion

### Design anomalies

Most of the studies in this review relied upon historical controls or comparison between clinical units with different patient population mixes. A before-and-after or one-size-fits-all design are not sufficiently reliable to present robust evidence for interventions aimed at controlling HAI [1, 12]. There were also many confounding practices, some of which were mentioned, or actively controlled and even discussed, but there were probably many more that were ignored and indeed, impossible to predict. The BETR study, in particular, attempted to control several potential confounders but failed to deliver incontrovertible results, which might have encouraged a plethora of linked publications [21, 24, 30, 63–65]. These were, perhaps, an attempt to justify implementation of a complex and no doubt costly sponsored study but despite controls and randomisation, adding UV to routine disinfection had little clinical impact, except possibly for VRE acquisition. An accompanying editorial emphasised the need for multimodal strategies for preventing HAI, particularly antimicrobial stewardship, since enhanced disinfection is only one piece of the puzzle [13]. Universal success in controlling healthcare pathogens with automated decontamination equipment is not necessarily guaranteed.

Another study questioned the lower-than-anticipated effectiveness of UV devices in eradicating *C. difficile* [50]. Since there was only < 50% removal of DAZO fluorescent gel during the mid-part of this study, it was thought that the cleaners had assumed superlative killing from the devices and relaxed their cleaning vigilance. Certainly, these devices are less effective at killing *C. difficile* spores in shaded areas. There was, however, an immediate and dramatic reduction of culture positive rooms during phase 3 of the study. Declining C. *difficile* from sampled surfaces was attributed not to the UV devices, but to the creation of a 3-person cleaning team, with daily disinfection of high risk sites using bleach, observed monitoring and supervisory sign off by the lead housekeeper [50].

### Comparisons between traditional cleaning and use of automated devices

There have been comparisons between traditional cleaning, with or without disinfectants, and decontamination using automated devices. Manual cleaning with bleach has been compared against several different disinfection methods, including H202, for terminal cleaning of hospital rooms contaminated with *C. difficile* spores [67]. Products were ranked according to log10 reductions in colony count from contamination to disinfection. While the most effective products were hydrogen peroxide, bleach (1000 ppm chlorine-releasing agent) and peracetic acid wipes, it was concluded that cheaper traditional methods using bleach were just as effective as modern systems. Comparative studies directly comparing disinfection modalities and cost benefits are limited [3, 67].

At least five studies compared routine terminal disinfection with UV devices [68–72]. Penno et al. described the effectiveness of a UV-C emitter in 22 hospital discharge rooms in a tertiary care academic hospital and compared it against terminal disinfection [72]. Using a cleanliness standard of < 5 cfu/cm^2^ for selected hand-touch sites, there were no differences between observed routine disinfection and use of UV-C. Previous studies have shown that non-covert observation of cleaners usually improves housekeeper disinfection [60, 61]. It is likely that carefully constructed standard operating procedures for cleaning staff, along with sufficient time, supervision and monitoring, represents the most cost effective strategy for protecting patients from HAI. Supervisors should tailor job requirements against staffing resources and cleaners should be supported, trained and adequately renumerated [2].

While patient and staff perceptions toward decontamination devices tend to be quite positive, the paucity of evidence for cost–benefit in this review challenges healthcare economists to recommend such technology for routine use [73]. This is not just because the devices are expensive. The equipment can generally only be used after the patient's discharge because patients and staff must vacate the room. However, near-patient sites constitute the highest risk as pathogen reservoirs and these need cleaning every day. [2] For ‘long stay’ patients, manual cleaning is the only option unless the patients are moved out of their rooms on a daily basis. This means that automated technology for room disinfection can only supplement, not replace, daily cleaning, which essentially means retention, rather than replacement, of the domestic workforce [74]. Thus, there is little opportunity for managers to off-set labour savings following device purchase, particularly when non-manual devices are unable to dispose of rubbish or deal with visible soil [11].

### Collateral damage

There are additional issues to consider for these devices. Despite initial eradication of surface flora in exposed areas, we know that surfaces are rapidly recolonised by environmental organisms within hours, including pathogens [75]. Secondly, the resources required to install, run and maintain these devices are considerable, even for hospitals in developed countries [14]. Low income countries might struggle to afford their use on a regular basis. Thirdly, the decontamination effect is not uniform, given that H202 cannot penetrate linen and soft furnishings and UV misses shaded areas. Neither product delivers expected outcome without first removing surface soil [11, 76].

There are further concerns over the long term impact of these devices, particularly if used on general wards rather than specialist units or when there is less risk of healthcare pathogen transmission. In common with all powerful disinfectants, they damage the environment in ways that we cannot always see. Both H202 and UV are toxic to people, pets and plants. [11] Adverse effects include the formation of high concentrations of hydroxyl and chlorine radicals, which encourage harmful reaction products when exposed to other chemicals found in indoor air [77]. Microbes themselves may survive noxious emissions from UV and H202 devices, which may be linked with emerging tolerance, resistance and cross-resistance among environmental pathogens [78–82]. For example, insufficient H202 would, as with bleach, activate microbial ‘SOS’ mechanisms, which encourage formation of new, or re-emergence of dormant, survival mechanisms. These facilitate DNA transfer to neighbouring organisms in a veritable shower of plasmid (and other genetic) exchanges coding for resistance to environmental assault [83].

UV light can cause bacterial mutations from a distance [81]. Laboratory trials of UV‐A and UV‐B exposure highlight the ability of microbial communities to enhance their radiation resistance over time if they are insufficiently exposed [84]. This suggests that resistance to UV‐C is highly likely without contained use and surveillance. Microbial communities adapt, reassemble, and persist, and recent theory in microbial ecology suggests that more gentle manipulation of the healthcare surface microbiome may be more sustainable than perpetual attempts at total removal [11, 82].

There is additional suspicion that introducing enhanced use of powerful disinfectants can release viable pathogens enmeshed in hard surface biofilm [85]. These organisms have been previously captured in microscopic crevices and their release reflects or even stimulates re-emergence of a previous outbreak [86, 87]. The biofilm lifestyles of microorganisms present a high risk for horizontal gene transfer, with transmission of antibiotic resistance and future recurrence [88]. Given these findings, routine use of microbiocidal products, including H202 and UV light, should be challenged [89].

### Universal standards and regulation

It has already been mentioned that there is no regulation of automated decontamination devices [79]. Chemical disinfectants used in the UK and Europe undergo stringent regulation by the European Chemicals Agency (ECHA) and by the Environmental Protection Agency (EPA) in the USA. Registration of a disinfectant against a given pathogen requires proof of efficacy using standardized test methods. Decontamination devices have not yet been regulated and consequently companies have engineered several different methods to demonstrate efficacy. This is less of a problem for hydrogen peroxide, because it has already been tested as a liquid disinfectant, albeit in different formulations to device-generated aerosol or vapour. UV technologies have not been standardized and this has created concern over in-use variations that have a substantial impact on measuring pathogen reduction, let alone any other effects. Factors such as shadowing; distance from UV source; targeted surface area; carrier orientation; and presence and type of organic material all affect the overall efficacy of UV devices [79, 90]. If these devices become commonplace for universal healthcare, they should undergo standardized testing to receive registration against different pathogens. This would provide consumers with a modicum of assurance that products are effective as well as encourage urgently needed cost–benefit evaluation. This is clearly in the interests of business and industry as well as healthcare.

## Conclusion

It was felt timely to independently review all available studies reporting an effect on HAI rates attributed to automated devices dispelling UV or H202. This systematic review is not just an assimilation of the clinical effects from these devices but a critical expose of the methods and pitfalls uncovered in the majority of studies reporting use. Device technologies offer a solution to just one aspect of infection prevention; this is because antimicrobial stewardship, isolation, hand hygiene and screening have already earned their place as useful strategies to control infection and all have been shown to reduce HAI rates [13, 91]. Doubtless there are yet more activities that can be added. It may be tempting to engage with modern technology when confronting HAI risks from environmental contamination, especially during an outbreak, but the findings in this review cannot support automated devices as a reliable alternative to manual and basic housekeeping practices. Current cleaning advice for occupied bed spaces, ‘One wipe; one site; and one direction’, with detergent and water, is easy, cheap and effective; and will not upset the surface ecology or create a futuristic ‘superbug’ [82, 92]. We should continue to support traditional infection control practices, including cleaning, without undue reliance on novel technology at the present time. [18].
